# Dynamics of *Campylobacter* colonization of a natural host, *Sturnus vulgaris* (European Starling)

**DOI:** 10.1111/j.1462-2920.2008.01773.x

**Published:** 2009-01

**Authors:** F M Colles, N D McCarthy, J C Howe, C L Devereux, A G Gosler, M C J Maiden

**Affiliations:** 1 The Peter Medawar Building for Pathogen Research, Department of Zoology, University of OxfordSouth Parks Road, Oxford OX1 3SY, UK; 2 The Edward Grey Institute of Field Ornithology, Department of Zoology, University of OxfordSouth Parks Road, Oxford OX1 3SY, UK

## Abstract

Wild European Starlings (*Sturnus vulgaris*) shed *Campylobacter* at high rates, suggesting that they may be a source of human and farm animal infection. A survey of *Campylobacter* shedding of 957 wild starlings was undertaken by culture of faecal specimens and genetic analysis of the campylobacters isolated: shedding rates were 30.6% for *Campylobacter jejuni*, 0.6% for *C. coli* and 6.3% for *C. lari.* Genotyping by multilocus sequence typing (MLST) and antigen sequence typing established that these bacteria were distinct from poultry or human disease isolates with the ST-177 and ST-682 clonal complexes possibly representing starling-adapted genotypes. There was seasonal variation in both shedding rate and genotypic diversity, both exhibiting a maximum during the late spring/early summer. Host age also affected *Campylobacter* shedding, which was higher in younger birds, and turnover was rapid with no evidence of cross-immunity among *Campylobacter* species or genotypes. In nestlings, *C. jejuni* shedding was evident from 9 days of age but siblings were not readily co-infected. The dynamics of *Campylobacter* infection of starlings differed from that observed in commercial poultry and consequently there was no evidence that wild starlings represent a major source of *Campylobacter* infections of food animals or humans.

## Introduction

*Campylobacter* is the most common bacterial cause of gastroenteritis worldwide and has an appreciable economic impact (Withington and Chambers, 1997; Allos, 2001; Roberts *et al*., 2003). The disease is usually self-limiting but more severe sequelae such as Guillain–Barré syndrome can occur (Altekruse *et al*., 1999; Allos, 2001). The major cause of human campylobacteriosis is *Campylobacter jejuni*, which accounts for approximately 90% of cases, with most of the remainder caused by *Campylobacter coli* (Gillespie *et al*., 2002). *Campylobacter lari* occasionally causes disease, mostly in immunocompromised patients (Tauxe *et al*., 1985; Martinot *et al*., 2001). Many risk factors for human infection have been identified, including the consumption of contaminated meat, particularly chicken, untreated water and unpasteurized milk (Frost, 2001; Baker *et al*., 2006). Members of the genus *Campylobacter* are readily isolated from animal and environmental reservoirs including farm animals and wild birds (Frost, 2001).

Epidemiological investigation of human campylobacteriosis has been particularly difficult due to the sporadic nature of the infection and difficulties with typing techniques. The advent of the high-throughput nucleotide based multilocus sequence typing (MLST) scheme for *Campylobacter* enables large-scale studies of *Campylobacter* in multiple hosts to be performed and compared worldwide, by providing definitive data that are directly comparable between host sources, accessible over the Internet and amenable to population genetic analyses (Dingle *et al*., 2001; Maiden, 2006). In addition to high-resolution typing, however, it is also necessary to study the population biology of diverse multihost bacteria such as *Campylobacter* in their natural reservoirs to understand the dynamics and sources of human infection (Gupta and Maiden, 2001; Maiden, 2006).

European Starlings (*Sturnus vulgaris*) are a potential source of infection for both humans and farm animals. They are commonly found both on farms and in towns and gardens, at times forming very large flocks leading to large-scale faecal contamination: a flock of 15 000 birds can cause more than 10^3^ defecations per square metre per night (Odermatt *et al*., 1998). Starlings have a relatively high carriage rate (40%) compared with some other wild bird taxa, with some evidence that genotyes similar to those isolated from human disease may be carried (Odermatt *et al*., 1998; Waldenstrom *et al*., 2002; Colles *et al*., 2003; Broman *et al*., 2004). High levels of colonization by *Campylobacter* among farmed poultry is a major problem faced by industry, and the study of populations among wild birds may provide insights into host interaction and environmental influences (Baker *et al*., 2006; Humphrey *et al*., 2007).

Here, the genetic diversity of 285 *C. jejuni* isolates obtained from the faeces of 957 wild starlings was examined by MLST. The nucleotide sequence of the *flaA* short variable region (SVR) was also determined, providing additional discrimination (Meinersmann *et al*., 1997; Dingle *et al*., 2002). Host factors such as age, weight and sex were recorded, together with the population dynamics of *C. jejuni* in recaptured birds and nest box colonies. Finally, any evidence that wild starlings may act as a source of human or farm animal infection was evaluated.

## Results

### Prevalence

**Fig. 1:**
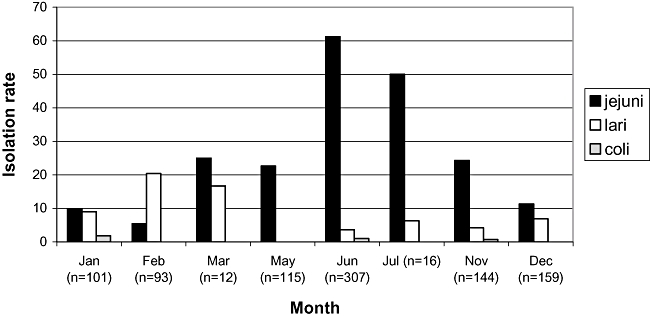
The isolation rates of *C. jejuni*, *C. coli* and *C. lari* during the course of a year. Samples were collected only in the months that are shown.

The overall isolation rate of *Campylobacter* species was 37.5% (359/957), with isolation rates of 30.6% (293 isolates) for *C. jejuni*, 0.6% (6 isolates) for *C. coli* and 6.3% (60 isolates) for *C. lari*. The prevalence of the *Campylobacter* species varied with month of the year, with *C. jejuni* being predominant during June and July, *C. lari* in February and March; *C. coli* was isolated though out the year in small numbers (Fig. 1). Logistic regression analysis using sine and cosine models indicated that the seasonal peak of *C. jejuni* was significant (*P* < 0.001) and that isolation rates did not differ significantly by year. Further analyses of *C. lari* and *C. coli* were outside the scope of this project.

### *Campylobacter* genotypes

Complete MLST data were obtained for 277 of the 285 *C. jejuni* isolates (97%), with 75 sequence types (STs) present which were assigned to 11 clonal complexes (Table 1). Twenty-five STs, accounting for 48 (16.4%) isolates, were unassigned to a clonal complex at the time of analysis. The most common clonal complex was the ST-682 complex, which accounted for 130 (44.4%) of isolates with 19 STs. The ST-177 complex was the second largest clonal complex present, accounting for 71 (24.2%) of the isolates with 16 STs. The remaining complexes accounted for less than 5% of isolates with fewer than four STs each.

**Table 1:** The *C. jejuni* genotypes isolated from wild European Starlings sampled in Oxfordshire in 2002–2005.

Clonal complex	ST	Frequency	Number of *flaA* SVR types
21	1383	1	1
42	42	1	1
45	45	5	3
	998	1	1
	1025	1	1
	334	1	1
48	38	2	1
177	177	46	10
	144	3	2
	563	2	2
	1004	3	1
	1014	1	1
	1382	2	2
	1482	1	1
	1500	1	1
	1506	1	1
	1535	1	1
	1381	2	1
	1485	1	1
	1533	1	1
	685	1	1
	1388	2	2
	1394	3	1
179	220	3	2
257	257	2	1
283	267	1	1
574	574	1	1
677	677	2	2
	1024	1	1
	1534	1	1
682	682	8	2
	1385	1	1
	1386	1	1
	1390	1	1
	1392	1	1
	1542	2	1
	686	15	4
	1019	1	1
	1020	63	10
	1021	4	2
	1022	13	2
	1027	13	4
	1391	1	1
	681	1	1
	687	1	1
	818	11	6
	1387	1	1
	1503	2	2
	1505	1	1
	1507	1	1
Unassigned	25 STs	48	23

**Fig. 2:**
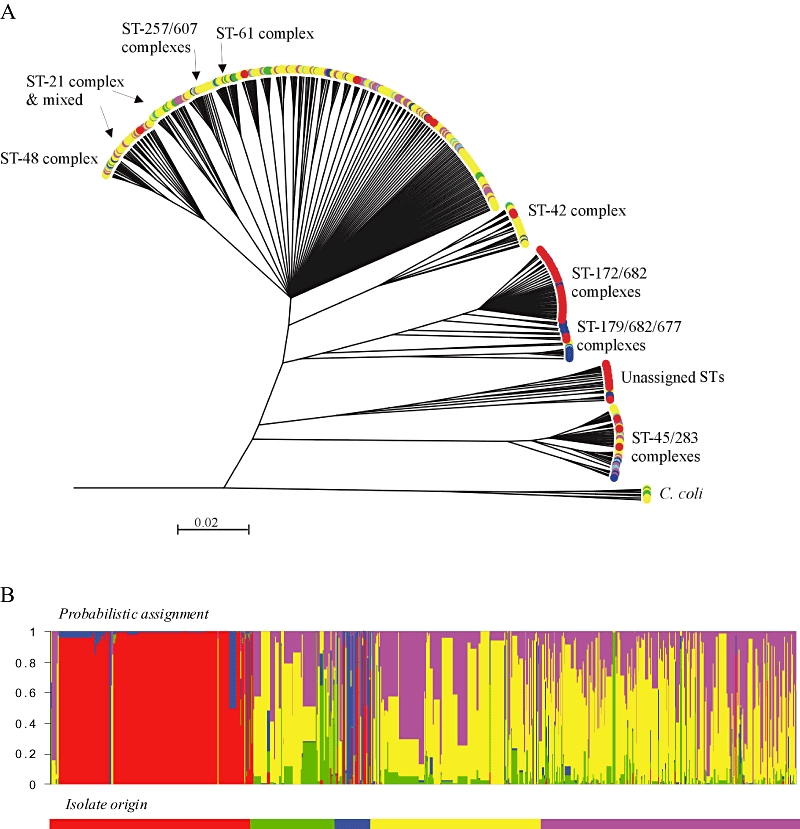
The distinct clustering of *C. jejuni* isolates from starlings. A. A Clonal Frame analysis demonstrating that the majority of *C. jejuni* isolates from starlings cluster separately from *C. jejuni* isolates representative of the diversity isolated from human disease and farm animals. Clusters that can be related to clonal complexes are indicated. B. The probabilistic assignment of the same *C. jejuni* allelic profiles to host source using Structure. Each allelic profile is represented by a vertical bar, showing the estimated probability that it comes from each of the sources identified. Key: starling (red), ruminant (green), environment (blue), human disease (yellow), poultry (pink).

The most common ST was ST-1020 (63 of isolates, 21.5%), followed by ST-177 (46 isolates, 15.7%). The remaining STs accounted for fewer isolates (< 5% each). Fifteen of the unassigned isolates grouped into small clusters sharing four or more alleles, but 10 were unrelated to each other (Table S1). In a genealogical analysis with Clonal Frame, the majority of *C. jejuni* isolates from starlings were clustered and distinct from *C. jejuni* representative of the diversity of genotypes isolated from human disease and farm animals (Fig. 2A). In addition, Structure analysis demonstrated that these genotypes showed strong host association with starlings (Fig. 2B) (Maiden and Dingle, 2008).

The majority of (124 of 142, 87.5%) of alleles from the starling isolates were shared with those from other sources. Of these, eight were associated with more than 75% starling and wild bird isolates, and 23 associated with more than 75% wild bird and environmental isolates on the *Campylobacter* MLST database. Eighteen alleles were unique to the study but they occurred at low frequency accounting for between one and five isolates.

### Genotype distribution over time

**Fig. 3:**
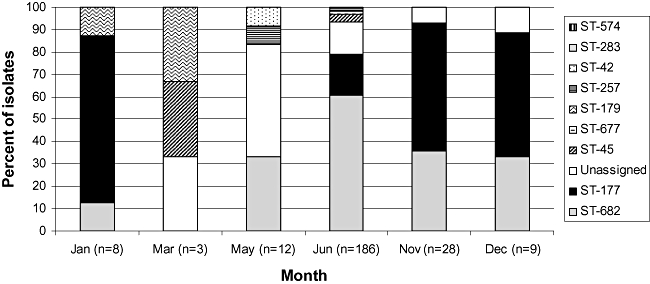
The *C. jejuni* clonal complexes isolated during 2004, by month. ST-574 and ST-283 were isolated in June and accounted for only 0.5% of the isolates each.

Of the 11 clonal complexes, six were identified in 2003 and nine were identified in 2004. Only four complexes (ST-682, ST-177, ST-45 and ST-179 complexes) were isolated in both years. The ST-682 complex was the dominant complex in both years accounting for 18.5% of isolates in 2003 and 53.2% of isolates in 2004. The ST-177 complex was the next most common and accounted for 14.8% of isolates in 2003 and 24.6% of isolates in 2004. The remaining complexes accounted for less than 8% of isolates each, in both 2003 and 2004. Analysis of clonal complex distribution in the year 2004 using Fisher's exact test gave evidence that the distribution of three of the clonal complexes [ST-179 complex (*P* < 0.0001), ST-177 complex (*P* < 0.0001) and ST-682 complex (*P* < 0.0001)] was not random over time (Fig. 3).

Seven of the 75 STs (ST-682, ST-1027, ST-818, ST-177, ST-1382, ST-45 and ST-179) were isolated in both 2003 and 2004, with ST-682 also isolated during 2002. The most commonly isolated ST was not necessarily the one isolated over the longest period of time; for example, ST-1020 was isolated 63 times over a time period of 35 days, but ST-257 was isolated on two occasions 369 days apart. The composition of the ST-682, ST-177, ST-45 and ST-179 complexes isolated over more than 1 month in 2004 varied with respect to ST. The complexes consisted of 34 STs between them, but only seven were isolated in more than 1 month. The Simpson's index of diversity of STs was 0.96 [confidence interval (CI) 0.92–0.99] in 2003 and 0.90 (CI 0.88–0.93) in 2004, where 1 indicates a diverse population and 0 indicates an identical population. The diversity of STs was calculated by month for 2004, recording values greater than 0.64 throughout the year, but with highest values recorded in May (0.95, CI 0.91–1) and June (0.88, CI 0.84–0.92) and the lowest in November (0.64, CI 0.52–0.75) and January (0.75, CI 0.52–0.98).

### Antigenic diversity

A total of 37 *flaA* SVR peptides and 54 *flaA* SVR alleles were detected among 285 *C. jejuni* isolates (Table 1 and Table S1). The association of *flaA* SVR type with both clonal complex and ST was not consistent. The greatest diversity was seen among the largest complexes, ST-682 and ST-177 complexes, having 19 and 16 different *flaA* SVR peptides respectively. In the ST most frequently isolated in 2004, ST-177, most of the associated *flaA* SVR types (19 of 29) were distinct, with only three *flaA* SVR types (124-68, 406-37 and 86-48) identified in more than 1 month. The remaining seven antigenic types were seen during 1 month only. Other STs were not isolated in sufficient numbers to investigate variation of the *flaA* SVR type over time.

### Carriage of *Campylobacter* among re-captured starlings

A total of 192 starlings were caught between two and nine times. From these birds, 199 *Campylobacter* isolates were obtained, of which 176 were *C. jejuni* and 23 *C. lari*. A total of 35 (18.2%) starlings were shedding *Campylobacter* species on each sampling occasion (time period between sampling from 1 to 588 days), 77 (40.1%) starlings were negative for *Campylobacter* species on each occasion (time period from 1 to 364 days), and 80 (41.7%) starlings changed between positive and negative status (time period between 1 and 392 days). *Campylobacter lari* was isolated from 16 (8.3%) of the re-captured starlings. Eight of these changed between shedding and not shedding *C. lari* (time period 6–519 days), three were shedding *C. lari* on each occasion sampled (time period from 1 to 20 days), and five changed between *C. lari* and *C. jejuni* (time period from 1 to 57 days).

The majority (31/37, 83.8%) of *C. jejuni* isolates from starlings positive for shedding on more than one occasion were a different genotype at different sampling times: in three birds different genotypes were obtained on consecutive days of sampling. Up to four different genotypes were isolated from an individual starling over a time period of 9 days. There were six instances where the same genotype was isolated from an individual starling more than once, two of which were sampled on consecutive days, three separated by 2 days and one separated by 5 days. Antigenic typing using the *flaA* locus revealed that four of the six birds carried the same ST-*flaA* SVR strain on two consecutive occasions. The remaining two birds carried the same ST on consecutive occasions separated by 2 and 5 days, but the *flaA* SVR had changed. The *flaA* SVR was unlikely to have changed by antigenic drift as the two types in each of the birds differed by 16 and 31 of 321 nucleotides, with polymorphic sites scattered throughout the region. Two days was the longest time period over which a *C. jejuni* strain with identical ST and *flaA* SVR type combination was isolated from the same bird.

### *Campylobacter* shedding among nestlings

A total of 20 samples in 2003 and 81 samples in 2004 were collected from nestling starlings when they were approximately 9 days of age. Of these, 12 (11.9%) were positive for *C. jejuni* with no other *Campylobacter* species isolated. Ten STs were identified and three clustered into the ST-42, ST-48 and ST-257 clonal complexes. Seven STs remained unassigned and had no more than three alleles in common with each other. Only one of the 10 STs from nestlings, ST-257, was isolated from the older starlings. Pair-wise *F*_ST_ comparing *C. jejuni* genotypes isolated from nestling and adult birds gave a value of 0.255 and *P*-value < 0.001, but nestling STs were scattered among the clusters of STs isolated from adults on a clonal fraMe tree (data not shown).

The 24 nests contained between two and six chicks, with an average of four chicks per nest. Nine of the 24 (37.5%) nests contained at least one chick shedding *C. jejuni*; seven contained only one chick shedding *C. jejuni*, one nest (nest 3, 2003) contained two of four chicks shedding *C. jejuni* and one nest (nest 32, 2004) contained at least three of four chicks shedding *C. jejuni*– no sample could be obtained from the fourth chick in the nest. The *C. jejuni* isolates from the positive siblings in nest 3 in 2003 had identical ST-*flaA* SVR alleles, and of the isolates from the three positive siblings in nest 32 in 2004, two had identical ST-*flaA* SVR alleles and the third had a different and unrelated ST. Two nestlings from 2003 were sampled at day 15: one, from a nest that contained no chicks shedding *Campylobacter* at 9 days was still negative at 15 days, while the other, from a nest with three nestlings that were shedding *Campylobacter*, was not sampled at day 9, but was negative at day 15.

The *flaA* SVR types isolated from nestlings were unusual, compared with those obtained from adult isolates, with eight of 10 being isolated from nestlings only, but they were distributed among the other *flaA* SVR types isolated from adult birds and did not form a monophyletic cluster on a Neighbour Joining Tree (data not shown). The distribution of *C. jejuni* positive nests and chicks was random with respect to location and year. An odds ratio (OR), calculated to determine if brood size was predictive of *C. jejuni* colonization, gave evidence to suggest a slight negative correlation, with smaller brood sizes being more likely to contain chicks shedding *C. jejuni* (OR 0.58).

### Correlation of *C. jejuni* shedding with bird age, weight, wing length and sex

As the age, wing length and weight variables were unlikely to be independent variables, logistical regression analysis was performed to determine which was the most influential. Age proved to be the most significant factor, with a *P*-value of < 0.001 (OR 0.50), although taking into account the variance in measurements, there was still some effect by weight (g) (OR 0.96, *P* = 0.108) and wing length (mm) (OR 0.94, *P* = 0.048). Specifically, juvenile birds (age code 3) were significantly associated with *C. jejuni* colonization (OR 1.69), with older birds becoming progressively less likely to be colonized.

Birds weighing less (an average weight of 75.2 g for birds with positive isolates and 79 g for birds that were negative), and with a smaller wing length (an average of 126.7 mm for birds with positive isolates and 129.9 mm for birds that were negative) were more likely to shed *C. jejuni* (*P* < 0.0001). With 95% confidence there was a 2.9–4.7 g difference in weight, and a 2.7–3.6 mm difference in wing length between birds that were shedding *C. jejuni* and birds that were not. There was no evidence that the sex of the bird affected *C. jejuni* status (*P* > 0.5).

## Discussion

Most studies of *Campylobacter* infection have concentrated on domestic birds, especially chickens. The aim of this study was to elucidate the population structure and dynamics of *Campylobacter* within wild European Starlings, in order that comparisons may be drawn with commercially reared poultry. Starlings were chosen as one of the most common and accessible species on the farm, with a high potential for interaction with the free-range chickens. The results indicate the dynamics and structure of *Campylobacter* among wild starlings are rather different to those described in farmed poultry.

Three thermophilic *Campylobacter* species, *C. jejuni*, *C. coli* and *C. lari*, were isolated from the wild starlings. The carriage rates of 30.6% *C. jejuni* and 6.3% *C. lari* were similar to rates recorded among other studies of mixed wild bird species (Kapperud and Rosef, 1983; Waldenstrom *et al*., 2002; Brown *et al*., 2004). The carriage of *C. coli* was low (0.6%) and erratic, being isolated in only 3 months of the most intensively sampled year, 2004. The results suggest the species does not frequently colonize starlings and is consistent with evidence that *C. coli* is more predominant among aquatic and marine birds (Waldenstrom *et al*., 2002).

There was marked seasonality in prevalence and diversity of *C. jejuni*, with the highest levels of both occurring during the late spring/early summer months. This observation was consistent with the peak in human disease in temperate countries, but reports of seasonality among wild birds vary (Broman *et al*., 2002; Waldenstrom *et al*., 2002; Sopwith *et al*., 2003). The incidence of human disease associated with bird-pecked milk has been reported to be highly seasonal, with the greatest numbers occurring in May and June (Sopwith *et al*., 2003). There is limited evidence of seasonal effects in prevalence among starlings, ducks, geese and blackbirds, while *Campylobacter* has been isolated most frequently from gulls in the autumn months (Broman *et al*., 2002; Waldenstrom *et al*., 2002). Consistent with the present study, small seasonal changes in genotypic diversity have been noted among wild geese (Colles *et al*., 2008a). The distribution of *C. jejuni* genotypes varied throughout the year, with ST-682 complex being more common in the summer and ST-177 complex being more common in the winter. These differences may be due to the different bird species, age groups, ecological guilds, food availability and sampling methods used (Waldenstrom *et al*., 2002). There was also some evidence of seasonality of *C. lari* shedding, with isolation more common in the winter months. This may be a consequence of the apparent ability for *C. lari* to survive for longer in surface waters compared with *C. jejuni* and *C. coli*, with length of survival being inversely correlated to the amount of sunlight (Obiri-Danso *et al*., 2001).

A strategy whereby single colonies were picked from a large number of faecal samples, rather than multiple colony picks from a small number of faecal samples was adopted in order to maximize the number of independent isolations thereby assessing the diversity more exhaustively and capturing rarer genotypes. The *C. jejuni* genotypes isolated were diverse, with many STs unassigned to clonal complexes; however, the closely related ST-682 and ST-177 complexes dominated in terms of frequency, number of genetic variants and temporal stability. Interrogation of the *Campylobacter* MLST database revealed that the majority of the STs belonging to these clonal complexes (81.3%) were isolated solely from starlings, with other host sources recorded rarely. These findings were supported by the Clonal Frame and Structure analyses where the majority of starling isolates clearly formed separate clusters from the known *C. jejuni* diversity identified in human disease and farm animals. While the ST-682 complex was prevalent among the starlings, the actual STs varied from year to year, perhaps through local clonal expansion. In particular, ST-1020 was common in June 2004 during a period when large numbers of birds were observed feeding on an open feed sack that became contaminated with bird faeces, possibly increasing the local transmission of this particular ST at that time. In contrast, within the ST-177 complex the central genotype predominated suggesting that this ST may represent a genotype that is particularly successful at surviving within starlings and their environment (Colegrave and Buckling, 2005).

Sequence typing of the *flaA* SVR was less discriminatory than MLST giving 54 alleles compared with 75 STs, but provided further differentiation of genotypes clustered with MLST. As seen in previous studies the antigen type was weakly associated with ST and clonal complex (Dingle *et al*., 2002). Certain *Campylobacter* strains, identified by identical ST and *flaA* SVR allele in combination, were stable over a period of at least 11 months. In contrast, other STs, for example, ST-177, were isolated in high frequency but the antigen typing data suggested that they originated from numerous different sources, rather than representing a single clone that was homogenous within the starling population.

Of the 192 birds captured on more than one occasion, equal proportions (40% and 42%) of birds were either negative for *Campylobacter* species on each occasion, or changed between positive and negative for shedding. Fewer (17.7%) were positive on each sampling occasion, but the time period between sampling was up to 392 days. There was no evidence that colonization of one *Campylobacter* species or genotype conferred immunity to another, or that single genotypes were carried over a long period of time. Instead there appeared to be a rapid replacement of genotypes, perhaps with some overlap as multiple carriage, although the study was not designed to investigate multiple carriage specifically. Other studies have reported a rapid turnover of colonizing strains in gulls, and urease positive thermophilic *Campylobacter* strains to be frequently acquired and lost by Redshank (Broman *et al*., 2002; Waldenstrom *et al*., 2007).

The presence of starling nest box colonies on the farm allowed the investigation of *Campylobacter* populations in nestling wild birds, approximately 9 days of age. Ten of the 81 (12.3%) nestlings sampled were positive for *C. jejuni*, but there was no evidence that a sibling was at any greater risk of acquiring the infection, despite being in close contact in the nest. Similarly there was some evidence that smaller broods were more likely to contain chicks shedding *Campylobacter*, although the sample size was small. The two starling-associated clonal complexes, ST-682 and ST-177 complexes, were not isolated and a marker of genetic differentiation (*F*_ST_) indicated that nestling genotypes were significantly different from those isolated from adult birds. The result was probably largely influenced by the fact that three STs commonly associated with human disease and farm animals were isolated from the nestlings. This may be a sampling effect or reflect differences in feed type, behaviour and susceptibility between nestling and adult birds (Moore, 1986; Kacelnik *et al*., 1995).

Of the host variables examined, age had the most significant effect on *Campylobacter* shedding. There was also some association of bird weight and, to a lesser extent, wing-length with *Campylobacter* shedding, although both factors were correlated with age. There is no evidence to date that *Campylobacter* colonization has any effect on the health of wild birds, although the smaller birds were more likely to be colonized by *Campylobacter*, which could reflect host immunity or general health status. Previous studies have indicated that diversity of *Campylobacter* may be significantly associated with flock growth rate and hock health among free-range broiler chickens and that hormones produced in response to stress may influence the onset of shedding (Cogan *et al*., 2007; Colles *et al*., 2008b). Some human studies have reported differences in the susceptibility of different sexes to *Campylobacter* but there appeared to be no effect of sex on *Campylobacter* carriage among the starlings (Louis *et al*., 2005). Similarly, Waldenstrom and colleagues (2002) found no effect of sex among several different species of wild bird.

Comparisons within the *Campylobacter* populations isolated from commercial chickens enabled inferences to be made of relevance to controlling infection on-farm. Early protection of poultry chicks, typically colonized at 21 days of age, is likely to be afforded by maternal antibodies or competing gut flora, but this does not appear to be the case with starling nestlings naturally infected as young as 9 days of age (Sahin *et al*., 2003). Adult starlings were less likely to be colonized by *Campylobacter* and there is some evidence that the same occurs in chickens (Newell and Fearnley, 2003). The most striking contrast is the absence of transmission between starling siblings in the same nest compared with the very rapid spread throughout a poultry flock within days of it being first detected (Newell and Fearnley, 2003). This perhaps reflects the practice by which parent starlings remove faecal sacs from the nest, while coprophagic behaviour of farmed poultry chicks may promote the rapid spread of *Campylobacter* throughout the flock (Willis *et al*., 2002). Diet influences the gut flora and natural immune defence mechanisms of poultry chicks and better health is likely to result from the more varied and natural diet experienced by wild starlings (Evans and Sayers, 2000; Newell and Fearnley, 2003). In addition stress levels, which in turn affect gut function, may differ considerably between the two bird species, with domestic poultry being kept in very large numbers and at high density (Humphrey, 2006). Starlings are only one of many wild bird species that could be tested, but the results from this study support other population studies of *C. jejuni* among wild birds, indicating that *C. jejuni* is highly host-specific with cross-transmission between different wild bird species occurring only rarely (Waldenstrom *et al*., 2007; Colles *et al*., 2008a)

In conclusion, wild starlings shed a diverse population of *Campylobacter* genotypes that is largely host-specific. The genotypes and patterns of transmission differ from those in farmed chickens, and further suggest that *Campylobacter* colonization in chickens is very strongly associated with their domestication. Consequently, this study provides no evidence to support the contention that wild starlings are a major source of infection of humans or farm animals.

## Experimental procedures

### Starlings

Wild starlings were trapped at the University farm at Wytham in Oxfordshire, UK, using mist nets along side animal pens and by whoosh nets and funnel traps baited with meal worms and chick feed. In addition, nestlings were sampled at approximately 9 and 15 days of age from nest box colonies at the farm and a second site, the sawmill, separated by approximately half a mile. Each bird was individually identified by a British Trust for Ornithology numbered leg ring, and the sex, age, weight and wing length (a standard measure of bird body size) recorded (Svensson, 1992; Gosler *et al*., 1998). The standard EURING codes based on markers such as plumage and feather length was used to age the birds: 1, nestling; 3, full-grown bird hatched in the present breeding season; 5, bird hatched in the previous calendar year; and 6, mature adult (Svensson, 1992).

### *Campylobacter* isolates

Faecal samples were collected from the cloth bags used to hold the birds which were autoclaved prior to use, and transferred to the laboratory using charcoal transport swabs. Most were cultured within 2 h of collection, but some were held at 4°C overnight, with no discernible reduction in isolation rate. The samples were cultured in 5 ml of Exeter broth (Nutrient broth No. 2, CM67, *Campylobacter* growth supplement SR084E, lysed defibrinated blood SR48, Oxoid, Basingstoke UK, Exeter selective supplement SV59, MAST group, Bootle, UK) at 37°C for 4 h and 42°C for 44 h. Putative *Campylobacter* isolates were subcultured onto mCCDA (PO0119A, Oxoid) and incubated microaerobically for a further 48 h at 42°C. Single colonies were subcultured to Columbia blood agar (PB0122A, Oxoid) and incubated microaerobically for 48 h at 42°C. *Campylobacter* colonies were identified by their appearance, Gram-negative curved rod morphology, and positive catalase and oxidase reactions. The ST or allele designations resulting from MLST were indicative of the *Campylobacter* species. DNA was extracted from the *Campylobacter* isolates using IsoQuick nucleic acid extraction kits (ISC Bioexpress, Kaysville, USA), following the protocol for rapid DNA extraction.

### Sequence typing

The published protocols for *Campylobacter* MLST and *flaA* SVR sequence typing were used (Meinersmann *et al*., 1997; Dingle *et al*., 2001; 2002). A combination of primers for PCR and sequencing from two different studies was found to give optimal results, with primers AspA8 5′-CTT CCA TGT GAG GAT TTA GC-3′ and AspA9 being a specific requirement for starling isolates (Dingle *et al*., 2001; Miller *et al*., 2005). The nucleotide extension reaction products were detected on an ABI Prism 3730 automated DNA analyser and assembled using methods described previously (Dingle *et al*., 2001). The consensus sequence was queried against the *Campylobacter* MLST database to give an allele number. The combination of seven allele numbers gave the ST. Sequence types were grouped into clonal complexes if they shared four or more alleles with the central genotype, using an automated script on the database. The unassigned isolates from the starling isolates and *Campylobacter* MLST database (http://pubmlst.org/campylobacter/) were analysed with the BURST algorithm to identify the central genotypes of potential new clonal complexes (Dingle *et al*., 2002; Feil *et al*., 2004). The central genotype is the predicted founding genotype from which others in the clonal complex have descended, and is identified by taking into account the number of single- and double-locus variants, rather than just being the most commonly isolated (Feil *et al*., 2004). A new complex was designated if it contained six or more STs, and the relevance confirmed by upgma cluster analysis (Sneath and Sokal, 1973; Dingle *et al*., 2002). The *flaA* SVR was sequenced and aligned using methods described previously (Meinersmann *et al*., 1997; Dingle *et al*., 2002). Other *Campylobacter* species were not further characterized.

### Genetic analysis

The pair-wise *F*_ST_ and test of significance calculations were performed using the arlequin software package version 3.0 (Wright, 1951; Schneider *et al*., 2000; Wilkinson-Herbots and Ettridge, 2004). A tool on the *Campylobacter* MLST database (http://pubmlst.org/campylobacter/) was used to prepare concatenated sequence giving a continuous nucleotide sequence of 3309 nucleotide pairs for each isolate, and data input files were prepared using the dnasp software package 4.0 (Rozas *et al*., 2003). An *F*_ST_ value of 0 indicated that the genotypes in two populations were identical, and a value of ±1 indicated they were distinct.

The genetic relationships between the STs isolated from starlings and those isolated from a diverse collection of human disease, ruminants, the environment (largely sand from bathing beaches) and poultry were analysed using Clonal Frame software (Dingle *et al*., 2002; Didelot and Falush, 2007). The model accounts for the fact that a single import event may result in changes at more than one nucleotide. Extended multi-FASTA files with concatenated sequence were prepared using a tool on the *Campylobacter* MLST database (http://pubmlst.org/campylobacter/). The tree was constructed using 50 000 burn-in cycles and 100 000 further iterations (Didelot and Falush, 2007).

The prediction of host origin using the same isolates as the Clonal Frame analysis was performed using Structure software (Pritchard *et al*., 2000; Falush *et al*., 2003; McCarthy *et al*., 2007). The data were input as allelic profiles and the PopData and PopFlag options used, so that isolates external to the training set were probabilistically assigned to a host population, based on their frequency within the host populations. The no-admixture model with lambda = 1 and independent allele frequency parameters were employed, with 10 000 burn-in cycles and 50 000 further replications for each analysis.

### Statistical analysis

A modified version of Simpsons' diversity index together with confidence intervals was used to determine typing diversity. A *D*-value of 1.0 indicated that each member of a population could be distinguished from every other, and a *D*-value of 0 indicated that all members of a population were identical (Hunter, 1990; Grundmann *et al*., 2001). The logistic regression analysis and test of significance was calculated using Stata (StataCorp LP, TX, USA). Comparison of *Campylobacter* shedding rates between years was considered to give a cyclic trend and thus sine and cosine lines were fitted as part of the analysis (Altman, 1991). A chi-squared test was performed to test whether or not the sine and cosine models were a good fit.

The odds ratio predictive of nest size and bird age and *Campylobacter* status, Student's *t*-tests comparing weight and wing length, Fisher's exact test assessing the distribution of clonal complexes during 2004 and chi-squared analysis comparing sex were calculated using Stata (StataCorp LP, TX, USA). Data from re-captured birds were removed from these analyses.

## Supporting information

**Table S1.** The *C. jejuni* genotypes isolated from wild European Starlings sampled in Oxfordshire in 2002–2005. u, untyped.
