# Supplementary material for: Dynamics of Campylobacter colonization of a natural host, Sturnus vulgaris (European Starling)
Source: Environ Microbiol. 2009 Jan;11(1):258–67. doi: 10.1111/j.1462-2920.2008.01773.x (PMC2702492; doi:10.1111/j.1462-2920.2008.01773.x)
Supplement: Supplementary file 1 [file emi0011-0258-SD1.doc]

Table S1. The *C. jejuni* genotypes isolated from wild European Starlings sampled in Oxfordshire in 2002-2005. u = untyped
